# Assessment of the MX3 hydration testing system in adults at rest and following exercise in the heat

**DOI:** 10.14814/phy2.70757

**Published:** 2026-03-26

**Authors:** Xiujing Zhao, Zachary J. McKenna, Rosie I. Perez, Shawn C. Wierick, Becca M. Allen, Brendon P. McDermott

**Affiliations:** ^1^ Heat & Hydration Optimization (H^2^O) Lab, University of Arkansas Fayetteville Arkansas USA; ^2^ Exercise Science Research Center University of Arkansas Fayetteville Arkansas USA; ^3^ Department of Health, Human Performance & Recreation University of Arkansas Fayetteville Arkansas USA

**Keywords:** exercise, heat, hydration, saliva osmolality

## Abstract

This study examined the performance of MX3 hydration testing system for assessing hydration status at rest and following exercise. Twenty adults completed two matched exercise trials in warm‐humid conditions. Pre‐ and post‐exercise saliva osmolality (S_osm_) were assessed using MX3. Reliability was assessed using mean difference (MD), absolute difference (ABD), coefficient of variation (CV), standard error of the measurement (SEM), intraclass correlation coefficient (ICC), and 95% limits of agreement (LoA). Diagnostic accuracy was assessed using a crosstab analysis relative to urine specific gravity (USG) and body mass loss (BML). Pre‐exercise, MX3 showed a MD of 4 mOsm/kg (95% LoA: −46, 54), ABD of 23 mOsm/kg, CV of 31%, SEM of 18 mOsm/kg, and ICC of 0.198. Post‐exercise, MX3 demonstrated a MD of 5 mOsm/kg (95% LoA: −78, 68), ABD of 28 mOsm/kg, CV of 29%, SEM of 26 mOsm/kg, and ICC of 0.391. Pre‐exercise, S_osm_ demonstrated a sensitivity of 0.09, specificity of 0.82, and LR^+^ = 0.51, LR^−^ = 1.11 relative to USG >1.020. Post‐exercise, S_osm_ showed a sensitivity of 0.57, specificity of 0.63, and LR^+^ = 1.52, LR^−^ = 0.69 relative to >2% BML. MX3 has low reliability and poor‐to‐weak predictive value in identifying hypohydration at rest and following exercise.

## 1 INTRODUCTION

Water is essential for maintaining overall health and physiological function, including circulatory regulation, metabolism, cellular homeostasis, and thermoregulation (Armstrong & Johnson, 2018). Water loss occurs continuously through the kidneys, lungs, gastrointestinal tract, and skin, and excessive loss can lead to inadequate hydration status (e.g., hypohydration) (Perrier et al., 2013). Even mild hypohydration (1%–2% body mass loss) can negatively affect health (El‐Sharkawy et al., 2015), cognitive function (Ganio et al., 2011), and exercise performance (Adams et al., 2019; Cheuvront et al., 2007). Therefore, accurate assessment of hydration status is crucial for guiding appropriate fluid replacement strategies and supporting optimal physiological function.

A variety of assessment techniques have been developed to evaluate hydration status across occupational, athletic, and military settings, including isotope dilution, neutron activation analysis, bioelectrical impedance, tracer appearance, plasma osmolality, urinary markers (e.g., urine specific gravity [USG], urine osmolality, and urine color), body mass loss, and saliva and tear osmolality (Armstrong, 2005). However, no single measure is sufficient to fully characterize hydration status due to substantial intra‐ and inter‐individual variability and methodological limitations (Francisco et al., 2025). Although plasma osmolality and blood urea nitrogen to creatinine ratio (BUN:Cr) are widely recognized as references to identify hydration status in laboratory and clinical settings (Armstrong, 2007; Fortes et al., 2015), their invasive and laboratory‐intensive nature limits their feasibility in field settings. As such, there remains a need for a reliable, noninvasive, portable, inexpensive, safe, and field‐appropriate hydration assessment.

Saliva osmolality (S_osm_), defined as the number of solute particles per kilogram of saliva, has emerged as a potentially biomarker capable of addressing several limitations of laboratory‐based assessments (Winter et al., 2024). S_osm_ has demonstrated reliability and validity for monitoring hydration changes in both sport (Muñoz et al., 2013; Oliver et al., 2008; Smith et al., 2012; Walsh, Laing, et al., 2004; Walsh, Montague, et al., 2004) and clinical settings (Fortes et al., 2015; Muñoz et al., 2013). For instance, S_osm_ strongly correlates with body mass changes and is as sensitive as urine osmolality for detecting hydration shifts during acute dehydration (Walsh, Laing, et al., 2004; Walsh, Montague, et al., 2004). Fortes et al. (2015) further found that S_osm_ provides superior diagnostic accuracy for identifying both water‐loss and water‐and‐solute‐loss dehydration compared with urine biomarkers and clinical signs. Despite this promise, relatively few field‐appropriate devices have been developed to track hydration change via S_osm_ measurement.

The MX3 hydration testing system, a handheld and portable osmometer, was recently developed for point‐of‐care, spot S_osm_ measurement. It has been used to evaluate hydration status in pediatric populations (Atjo et al., 2022) and in hypertensive patients (Faidah et al., 2021). However, its reliability has not been thoroughly established, although preliminary evidence suggests moderate‐to‐good reliability (Atjo et al., 2022; Faidah et al., 2021; Winter et al., 2024). Moreover, while the MX3 hydration testing system has shown diagnostic accuracy for routine hydration screening in clinical settings (Atjo et al., 2022; Faidah et al., 2021), its performance in sport settings, particularly following exercise, remains insufficiently explored. Therefore, the present study aimed to examine the MX3 hydration testing system at rest and following a bout of self‐paced exercise in warm‐humid conditions.

## 2 METHODS

### 2.1 Subjects

These data are part of a larger project investigating the validity and reliability of various sweat assessment techniques. Twenty healthy, active (defined as exercise at least 3 times a week) adults (10 males and 10 females) took part in this study. Participants completed both trials between May and June in the mid‐South of the United States (21.1°C, 70% relative humidity, Climate‐Data.org) and were assumed partially heat‐acclimatized (Brown et al., 2022). Participant characteristics are presented in Table 1. Prior to recruitment, this study was approved by the University of Arkansas Institutional Review Board (IRB#: 2502587094) in April 2025. All participants provided written informed consent and study procedures were thoroughly explained, before participation.

**TABLE 1:** Participant characteristics (*n* = 20).

Characteristics	Means ± SD [min, max]
Age (y)	33 ± 11 [20,47]
Height (m)	1.70 ± 0.11 [1.56, 1.92]
Mass (kg)	71.1 ± 14.8 [46.6, 97.6]
Body mass index (kg/m^2^)	24.1 ± 2.8 [20.0, 29.2]
Body surface area (m^2^)	1.83 ± 0.24 [1.46, 2.25]

### 2.2 Experimental procedures

Each participant completed two matched, self‐paced, 60‐minute cycling or running trials, separated by 2 days, in warm‐humid conditions (27.3 ± 0.4°C, 50.9 ± 1.8% relative humidity, 22.4 ± 0.2°C wet‐bulb globe temperature [WBGT]). Prior to each trial, participants were provided with diet and fluid logs to record food and drink intake for 24 h. The participants were asked to match their diets the day before each trial. Upon arrival, participants provided a spot urine sample to verify euhydration, defined as urine‐specific gravity ≤1.020 (USG, Master‐SUR, Atago L td, Tokyo, Japan) (Armstrong, 2007). If USG was >1.020, participants were provided with 500 mL of water to consume 30 minutes before the commencement of exercise.

After pre‐exercise saliva samples (described in detail below) and nude body mass were obtained, participants performed self‐selected moderate‐intensity 60 min of either treadmill running (F63, SOLE Manufacturing Inc., Jonesboro, AR, USA, or PRO‐FORM, ICON Health & Fitness, Inc., West Logan, UT, USA, *n* = 10) or cycling (Velotron, Racermate Inc.; Seattle, WA, USA, *n* = 10). No fluids were provided during exercise to prevent the potential of confounding effects of oral intake on S_osm_ (Ely et al., 2011). Heart rate (Timex Group, Middlebury, CT), rectal temperature (RET‐1, Physitemp Instrumental Inc., Clifton, NJ, USA), and the 6–20 scale rating of perceived exertion (Borg, 1970) were recorded at 15‐min intervals throughout the trial. Following the completion of trials, post‐exercise saliva samples and nude body mass were collected.

### 2.3 Hydration measurements

#### 2.3.1 Saliva osmolality (S_osm_)

Pre‐ and post‐exercise S_osm_ were assessed using the MX3 hydration testing system (MX3 Diagnostics, Inc., Minneapolis, MN, USA). The MX3 hydration testing system is a portable (~160 g, 214 mm × 45 mm × 25 mm), battery operated (Type Single Cell rechargeable, Li‐Po, 3.7 V, 1100mAh) point‐of‐care analyzer that uses disposable pre‐calibrated biosensors to determine S_osm_ based on electrochemical impedance spectroscopy, with quantitative digital output of S_osm_. S_osm_ was measured by tapping the pre‐calibrated biosensor strip directly on the participants' tongue for several seconds until the osmometer registered that a sample had been collected (according to manufacturer recommendations). Sampling was performed for all participants. Saliva sampling was performed with the prerequisite of no eating or drinking 30 min prior to the measurement. Subjects were categorized as hydrated if S_osm_ was ≤65 mOsm/kg, mildly dehydrated if S_osm_ was 65–100 mOsm/kg, moderately dehydrated if S_osm_ was 101–150 mOsm/kg, or severely dehydrated if S_osm_ was ≥151 mOsm/kg.

#### 2.3.2 Urine specific gravity (USG)

The test was conducted as a spot‐check performed prior to the 60‐min exercise in the chamber. Upon arrival, each subject provided a spot urine sample. Researchers then immediately measured USG using a handheld clinical refractometer (Master‐SUR, Atago L td, Tokyo, Japan). Using the USG of 1.020 as the threshold of hypohydration and euhydration (Armstrong, 2007).

#### 2.3.3 Body mass loss

Body mass loss was calculated from the change in nude body mass from pre‐ to post‐exercise, corrected for urine loss. The American College of Sports Medicine (Cheuvront et al., 2007) and the National Athletic Trainers' Association (McDermott et al., 2017) suggest fluid replacement to limit hypohydration to <2% of body mass loss. Therefore, we used a body mass loss of 2% as the threshold between hypohydration and euhydration.

### 2.4 Statistical analysis

Statistical analyses were conducted using RStudio (4.4.1) or GraphPad Prism (Version 10.2.3). Descriptive characteristics were calculated for all study variables and reported as mean ± standard deviation (SD). The pre‐ and post‐exercise reliability of the MX3 hydration testing system was evaluated using values from each trial and determined using the mean difference (MD), absolute difference (ABD), coefficient of variation (CV), standard error of measurement (SEM), intraclass correlation coefficient (ICC), and 95% limit of agreement (LoA). A two‐way analysis of variance was used to compare differences between pre‐ and post‐exercise S_osm_ across trials 1 and 2. The CV was interpreted as: very good (<10%), good (10%–20%), acceptable (20%–30%), and not acceptable (>30%) (Walters et al., 2021). The ICC was interpreted as: poor (<0.50), moderate (0.50–0.75), substantial (0.75–0.90), and almost perfect (>0.90) (Koo & Li, 2016).

To determine the ability of the MX3 hydration testing system in identifying hydration status, a standard crosstab analysis with likelihood ratio calculations was conducted to assess whether pre‐ and post‐exercise S_osm_ predict USG at rest and body mass loss following exercise. Prior to conducting predictive analyses, the cut‐point of S_osm_, USG, and body mass loss percentage were 65 mOsm/kg, 1.020, and 2%, respectively. Statistical significance was set α priori at *p* ≤ 0.05.

## 3 RESULTS

Twenty healthy, active, partial‐acclimatized adults (10 females) completed two matched, self‐paced 60‐minute cycling (*n* = 10, 149 ± 72 watts) or running (*n* = 10, 11.3 ± 1.6 km/h) trials in warm‐humid conditions. Prior to exercise, USG was measured to verify euhydration (≤1.020) (Armstrong, 2007), and participants with USG >1.020 were provided 500 mL of water to standardize hydration status. Pre‐ and post‐exercise S_osm_ was measured using the MX3 hydration testing system under controlled conditions, including consistent biosensor placement, standardized exercise intensity and environment.

Participant characteristics are shown in Table 1. No significant differences were found in USG (trial 1: 1.010 ± 0.007; trial 2: 1.010 ± 0.007, *p* = 0.732), body mass loss (trial 1: 1.06 ± 0.38; trial 2: 1.05 ± 0.40 kg, *p* = 0.934), peak T_re_ (trial 1: 38.5 ± 0.5°C; trial 2: 38.6 ± 0.5°C, *p* = 0.316), peak heart rate (trial 1: 147 ± 14 bmp; trial 2: 147 ± 14 bpm, *p* = 0.913), or peak RPE (trial 1: 12 ± 2; trial 2: 12 ± 2, *p* = 0.871) between trials. For environmental conditions, there were no differences in WBGT (*p* = 0.848), ambient temperature (*p* = 0.526), and relative humidity (*p* = 0.118) between trials.

**FIGURE 1:**
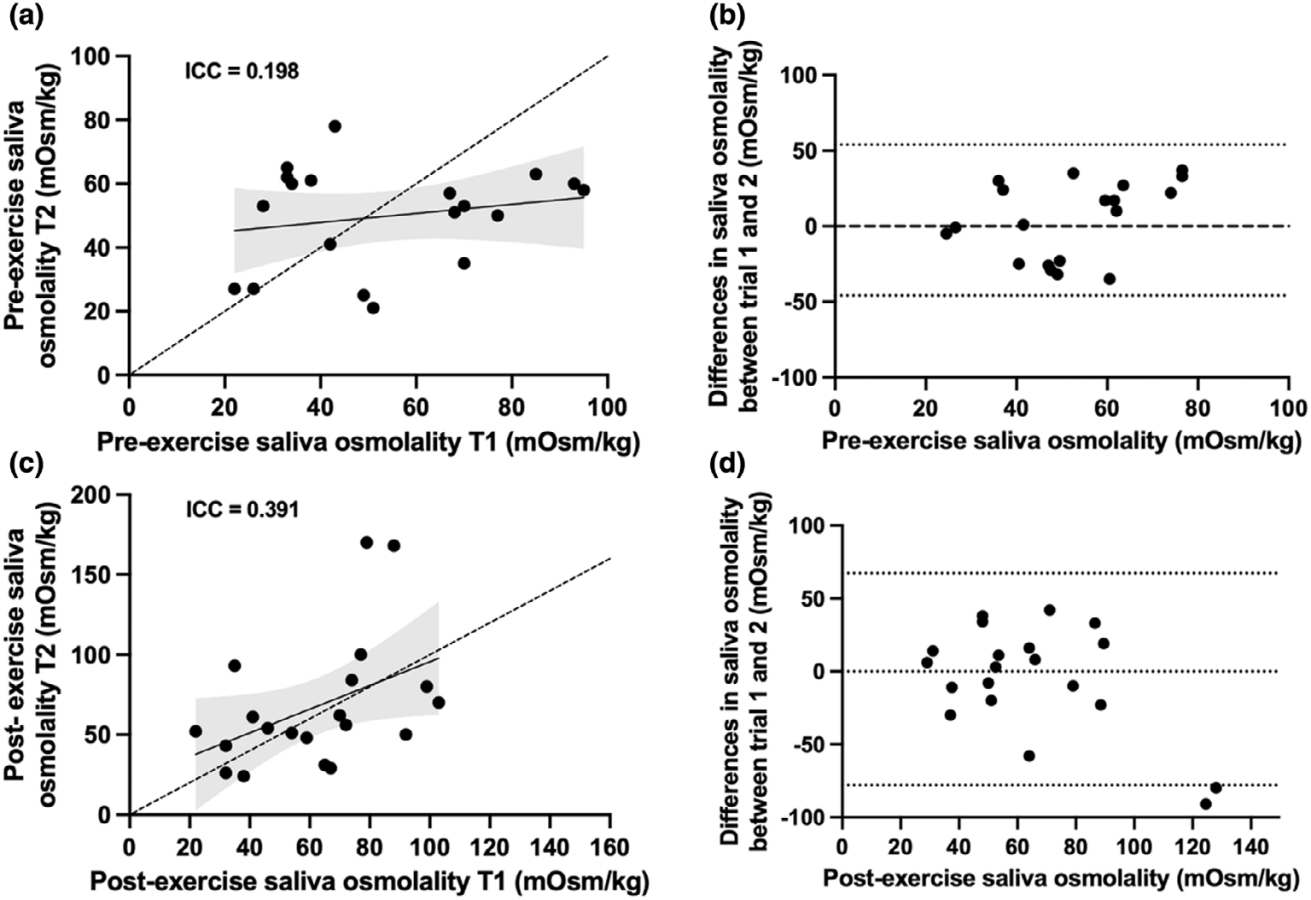
Reliability analysis of the MX3 hydration testing system for measuring saliva osmolality. (a) Intraclass correlation (ICC) plot between the first and second trials from MX3 pre‐exercise assessment (dashed line represents the line of identity); (b) Bland–Altman plot and 95% limits of agreement (thin black dashed lines) of the first and second trials from MX3 pre‐exercise assessment; (c) Correlation plot between the first and second trials from MX3 post‐exercise assessment; (d) Bland–Altman plot and 95% limits of agreement of the first and second trials from MX3 post‐exercise assessment.

**FIGURE 2:**
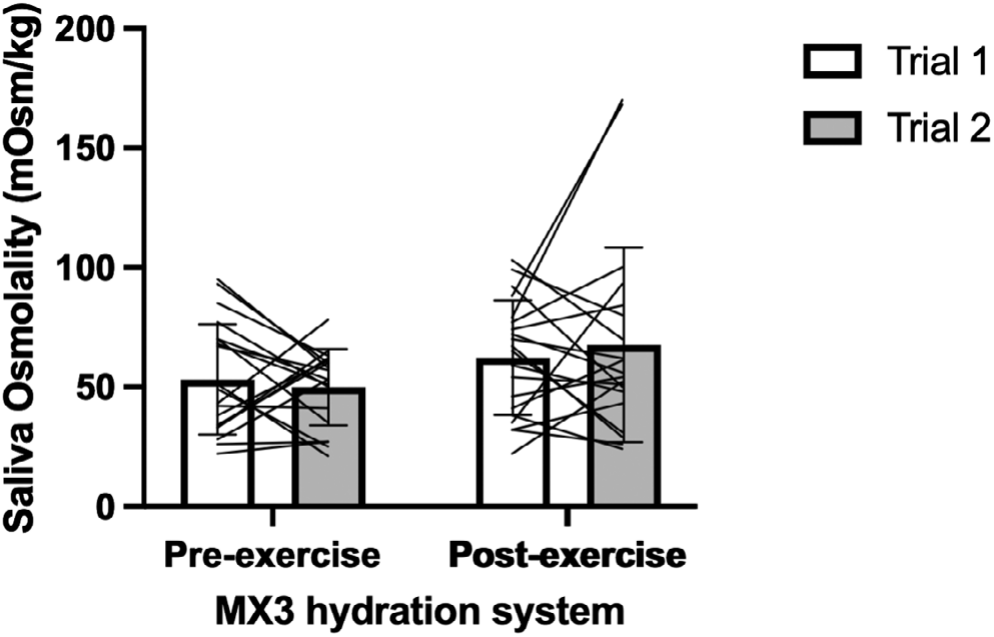
Differences in saliva osmolality between pre‐exercise and post‐exercise across the two trials.

The reliability for the MX3 hydration testing system is presented in Table 2, Figures 1 and 2. There was a main effect of exercise (pre‐ vs. post‐ exercise: *p* = 0.028), but no main effect of trial (*p* = 0.873) or exercise by trial interaction (*p* = 0.384) for S_osm_ measured by MX3. At rest, MX3 exhibited a MD of 4 mOsm/kg (95% LoA: −46, 54), ABD of 23 mOsm/kg, CV of 31%, SEM of 18 mOsm/kg, and ICC of 0.198. Following exercise, the MX3 hydration testing system demonstrated a MD of 5 mOsm/kg (95% LoA: −78, 68), ABD of 28 mOsm/kg, CV of 29%, SEM of 26 mOsm/kg, and ICC of 0.391, indicating inconsistent measurements across repeated trials.

**TABLE 2:** Reliability of the MX3 hydration testing system for measuring saliva osmolality.

Variable	MX3 pre‐exercise		MX3 post‐exercise	
	Trial 1	Trial 2	Trial 1	Trial 2
No. of samples	20	20	20	20
Mean ± SD (mOsm/kg)	53 ± 23	50 ± 16	62 ± 24	68 ± 41
Range (mOsm/kg)	22–95	21–78	22–103	24–170
Mean difference (mOsm/kg)	4		5	
ABD (mOsm/kg)	23		28	
95% LoA	− 46, 54		− 78, 67	
SEM (mOsm/kg)	18		26	
CV (%)	31		29	
ICC	0.198		0.391	

Abbreviations: ABD, absolute difference; CV, coefficient of variation; ICC, interclass correlation coefficient; LoA, Limits of Agreement; SEM, standard error of measurement; SD, standard deviation.

Table 3 shows the predictive analysis for the MX3 hydration testing system. Across all trials (*n* = 40), six trials had pre‐exercise USG >1.020, and five trials exceeded >2% body mass loss following exercise, indicating hypohydration. At rest, the sensitivity and specificity of S_osm_ for identifying hydration status based on USG were 0.09 and 0.82, respectively. Following exercise, the sensitivity and specificity of S_osm_ for predicting body mass loss‐defined hydration status were 0.57 and 0.62, respectively. These results illustrate the MX3's limited ability to detect dehydration changes both at rest and following exercise in the heat.

**TABLE 3:** Predictive analysis for urine specific gravity (USG, 1.020) and pre‐exercise MX3 saliva osmolality (S_osm_, 65 mOsm/kg), body mass change (BML, 2%), and post‐exercise MX3 saliva osmolality (S_osm_, 65 mOsm/kg).

Variable	USG vs. pre‐MX3 S_osm_	BML vs. post‐MX3 S_osm_
Sensitivity	0.09 (CI: 0.02–0.38)	0.57 (CI: 0.25–0.84)
Specificity	0.82 (CI: 0.64–0.92)	0.63 (CI: 0.45–0.77)
LR+	0.51 (CI: 0.07–3.88)	1.52 (CI: 0.70–3.33)
LR‐	1.11 (CI: 0.86–1.43)	0.69 (CI: 0.28–1.68)

*Note*: LR+ positive likelihood ratio; LR− negative likelihood ratio.

This application demonstrates how MX3 can be used to monitor physiological hydration status in a controlled exercise research setting, though with low reliability and weak predictive accuracy under the conditions tested.

## 4 DISCUSSION

The present study aimed to examine the MX3 hydration testing system at rest and following exercise during two matched, self‐paced 60‐min cycling or running trials in warm‐humid conditions. To our knowledge, this is the first study to evaluate MX3 at rest and following exercise in the heat. Several key findings should be highlighted. First, the MX3 hydration testing system demonstrated low between‐trial reliability S_osm_ both at rest and following exercise. Second, compared with USG at rest, MX3 showed very poor predictive ability for hypohydration. While the analyzer performed slightly better following exercise, compared with body mass loss, the diagnostic accuracy for detecting hypohydration remained weak. Collectively, these findings indicate that MX3 has low reliability S_osm_ and shows poor‐to‐weak predictive value for identifying hypohydration at rest and following a 60‐min self‐paced exercise in the heat.

Reliability of an instrument is essential for longitudinal monitoring of hydration status and making informed decisions regarding fluid intake. Given that S_osm_ exhibits greater intra‐individual variability than USG and plasma osmolality (Cheuvront et al., 2010; Ely et al., 2011), evaluating the reliability of the MX3 hydration testing system for measuring S_osm_ under varying conditions is important. Using multiple statistical approaches, we found that pre‐exercise S_osm_ by the MX3 hydration testing system yielded “not acceptable” results (Walters et al., 2021), and a “poor” ICC (Koo & Li, 2016). Following the 60‐min exercise bout, reliability remined low. Although the CV value improved slightly and falls within an “acceptable” range (Koo & Li, 2016), the ICC remained “poor” (Walters et al., 2021). Overall, these findings suggest that MX3 provides inconsistent S_osm_ measurement across trials, both at rest and following exercise. These findings are inconsistent with a previous study reporting moderate‐to‐good intra‐day, test–retest reliability for the MX3 hydration testing system in the assessment of S_osm_ (Winter et al., 2024). However, there are clear methodological differences that likely explain this discrepancy. For example, Winter et al. (2024) used two saliva samples collected ~3–5 minutes apart, this design may be appropriate for evaluating instrument error but not for true day‐to‐day use, as it inherently minimizes biological variation and inflates reliability estimates. In contrast, trials in the current study were separated by a 2‐day test–retest interval. Even though exercise intensity, fluid intake, and environmental conditions were matched between trials, biological changes in S_osm_ over 48 h are expected and likely contributed to the large variability observed. As such, the present findings may better reflect real‐world variability when S_osm_ is used to monitor day‐to‐day hydration status.

Beyond reliability, the ability of the MX3 hydration testing system to detect actual changes in hydration status is also critical. At rest, S_osm_ measured by MX3 demonstrated very low sensitivity (0.09) but moderate specificity (0.82) compared to hypohydration defined by USG >1.020. This shows that MX3 has poor ability to detect hypohydration, but reasonable accuracy in identifying euhydration. Following exercise, S_osm_ measured by MX3 demonstrated moderate sensitivity (0.57) and specificity (0.63) relative to >2% body mass loss. However, our likelihood ratios indicate weak diagnostic accuracy, suggesting limited usefulness of the MX3 hydration testing system for detecting exercise‐induced hypohydration. Therefore, MX3 demonstrated a poor‐to‐week predictive accuracy for hypohydration both at rest and following exercise. Two previous studies conducted in clinical settings reported stronger accuracy for MX3 (Atjo et al., 2022; Faidah et al., 2021). These studies used different S_osm_ and USG thresholds in defining hydration status. For example, Atjo et al. (2022) reported high sensitivity (0.786–0.865) and specificity (0.867–0.91.1), with a LR^+^ of 6.5–8.8, using S_osm_ cut point of 77 mOsm/kg and USG threshold of >1.025, or >1.030, indicating an ability to detect hypohydration. Faidah et al. (2021) similarly reported that the MX3 hydration testing system has strong discrimination capacity for the identification of highly dehydrated individuals, with a sensitivity and specificity of 81.82% and 64.06%, respectively, using a cut‐point value of USG (≥1.030) and S_osm_ (≥70.5 mOsm/kg), respectively. Differences in classification thresholds likely account for the discrepancy between these studies and present findings. Further, although two unpublished reports by MX3 Diagnostics, Inc. suggest the MX3 hydration testing system may be valid compared with the two laboratory‐based osmometers (Osmette III, Precision Systems Inc., Calumet City, IL; Model 3320, Advanced Instruments, Norwood, MA) (Kwan, 2019; Pranavmurthi et al., 2020), formal validation under controlled research conditions remains limited and should be addressed in future studies.

This study is not without limitation. First, the relatively small sample of active, healthy, partially heat‐acclimated individuals in the present study limits statistical power and results may not generalize to broader or less acclimated populations. Second, our data included relatively few participants who had USG >1.020 (6/40 trials) or BML >2% (5/40 trials), which likely reduced the ability to adequately evaluate the MX3 hydration testing system's capacity to identify true positives and negatives. Third, this study used an exercise model consisting of a 1‐h, self‐selected, moderate‐intensity exercise bout performed under warm‐humid conditions. The findings derived from this model may not reflect longer duration exercise or other environmental scenarios. Fourth, the self‐selected exercise intensity could have introduced variability in workload and physiological responses, although our physiological data indicate matched exercise. Finally, the present study did not include a validity assessment comparing MX3 hydration testing system with a laboratory‐based osmometer for S_osm_. Future studies should investigate the reliability and validity of the MX3 hydration testing system across longer exercise duration, wider environmental conditions, standardized exercise intensities, and a larger sample size.

## AUTHOR CONTRIBUTIONS

B.P.M.: Supervision, conceived the study, writing–review and editing; X.Z.: Conceived the study, writing‐original draft; B.P.M., Z.M., and X.Z.: Analyzed the data; Z.M., S.C.W., R.I.P, and B.M.A.: Writing–review and editing. All authors have read and agreed to the published version of the manuscript.

## FUNDING INFORMATION

This study was funded by hDrop, Inc., which had no role in the study design, data collection, data analysis, interpretation of the results, manuscript preparation, or conclusions. MX3 provided equipment and devices used in the study but had no involvement in the study design, data collection, data analysis, interpretation of the results, manuscript preparation, or conclusions.

## CONFLICT OF INTEREST STATEMENT

The authors have no conflict of interest to declare.

## Data Availability

The data present in this study are available on reasonable request from the corresponding author.
